# Wildfire spread, hazard and exposure metric raster grids for central Catalonia

**DOI:** 10.1016/j.dib.2017.12.069

**Published:** 2018-01-04

**Authors:** Fermín J. Alcasena, Alan A. Ager, Michele Salis, Michelle A. Day, Cristina Vega-Garcia

**Affiliations:** a Agriculture and Forest Engineering Department (EAGROF), University of Lleida, Alcalde Rovira Roure 191, 25198 Lleida, Catalonia, Spain; b USDA Forest Service, Pacific Northwest Research Station, Western Wildland Environmental Threat Assessment Center, 3160 NE 3rd Street, Prineville, OR 97754, USA; c National Research Council, Institute of Biometeorology (CNR-IBIMET), Regione Baldinca, 07100 Sassari, Italy; d Euro-Mediterranean Center on Climate Change (CMCC), IAFES Division, Via Enrico De Nicola 9, 07100 Sassari, Italy; e Oregon State University, College of Forestry, Forest Ecosystems & Society, 321 Richardson Hall, Corvallis, OR 97331, USA; f Forest Sciences Centre of Catalonia, Carretera de Sant Llorenç de Morunys km 2, Solsona 25280, Catalonia, Spain

**Keywords:** Catalonia, Wildfire exposure, Fire transmission, Crown fire activity, Prescribed fires

## Abstract

We provide 40 m resolution wildfire spread, hazard and exposure metric raster grids for the 0.13 million ha fire-prone Bages County in central Catalonia (northeastern Spain) corresponding to node influence grid (NIG), crown fraction burned (CFB) and fire transmission to residential houses (TR). Fire spread and behavior data (NIG, CFB and fire perimeters) were generated with fire simulation modeling considering wildfire season extreme fire weather conditions (97^th^ percentile). Moreover, CFB was also generated for prescribed fire (Rx) mild weather conditions. The TR smoothed grid was obtained with a geospatial analysis considering large fire perimeters and individual residential structures located within the study area. We made these raster grids available to assist in the optimization of wildfire risk management plans within the study area and to help mitigate potential losses from catastrophic events.

**Specifications Table**

**Table:** 

Subject area	Environmental sciences, forestry.
More specific subject area	Natural hazards
Type of data	Maps (×4)
How data was acquired	Fire simulation modeling and a geospatial analysis with a geographic information system (GIS).
Data format	Raster grids at 40 m resolution (.tif).
Experimental factors	Extreme fire weather conditions in terms of fuel moisture content and wind speed for the wildfire season dominant scenario (southern wind) were considered to model wildfire spread and behavior.
	We only considered residential houses within the study area for the transmission analysis, excluding industrial areas, farms and any other structures.
	Modeling output fire perimeters < 100 ha were excluded from the transmission analysis.
Experimental features	We used FlamMap for wildfire spread and behavior modeling (Finney 2006) and geographic information system software to conduct the transmission and geospatial analysis (ArcMap version 10.1). ArcFuels was used to create ensemble landscape input data for fire modeling (Ager et al., 2011), and the Fire Family Plus program was used to process weather data (Bradshaw and McCormick, 2000).
Data source location	All the landscape file fire modeling input data (topography, surface fuels and canopy metrics) corresponded to the Bages County in central Catalonia (northeastern Spain) plus a 6 km buffer.
	We used hourly meteorological data (1998 to 2016 records) from the Castellnou de Bages automatic weather station (U4 station reference, Longitude 1.832°N and Latitude 41.842°E) to characterize the fire weather modeling scenario.
Data accessibility	The repository of the University of Lleida (**URL**): http://hdl.handle.net/10459.1/60357
Related research article	Alcasena FJ, Ager AA, Salis M, Day MA, Vega-Garcia C. Otimizing prescribed fire allocation for managing fire risk in central Catalonia. Sci Total Environ. 2018 4:872-885.

**Value of the Data**

• We provide spatially-explicit value grids for major wildfire risk causative factors in Bages County, central Catalonia (northeastern Spain).
• The raster grids provide quantitative value data to assist ongoing fuels management programs aiming at reducing wildfire risk efficiently.
• The node influence grid (NIG) identifies high fire activity cells or pixels on the landscape (strategic areas) where fuel treatments restrict large fire potential.
• The crown fraction burned (CFB) grid provides data on wildfire effects to the overstory, related to tree mortality and crown fire activity. We generated CFB grids fore extreme fire weather and prescribed fire conditions. The data provide valuable information to prescribe fuel treatments on forested areas.
• Fire transmission to residential houses (TR) provides quantitative exposure data for large fires spreading long distances across the landscape and defines the scale of risk to communities.

## 1 Data

The Node Influence Grid (NIG) is a raster output that quantifies for each pixel the number of nodes in fire-flow direction from that cell onwards exerting influence on the fire pathways, as the logarithm (ln) of the number of nodes burning as a result of burning through that particular node [2]. The higher the frequency, the stronger the influence is on fire pathways. The log-transform is required to facilitate the visualization of details, since the node counts can range over 4 or 5 orders of magnitude (e.g. from 1 to 10^4^). Node influence is highly dependent on fire weather conditions (i.e., wind speed, wind direction and fuel moisture content) and the arrangement of fuel on the landscape. Details of the geospatial data:

– Node influence grid for extreme fire weather scenario (**NIG.tif**). Units= ln of number of nodes. Resolution= 40 m. Coordinate system= ETRS89 UTM 31N.

Crown Fraction Burned (CFB) indicates the degree of potential crown fuel consumption as a proportion of the total number of tree crowns (fraction between 0 and 1), and indicates the probable type of fire activity [1]. The fire types can range from surface fire (< 0.10) to continuous crown fire (> 0.90), while intermediate values represent a scaled value of intermittent crown fire. Crown fire activity is calculated independently of any neighboring cells, and does not consider fire front spreading direction in the calculations. Details of the geospatial data:

– Crown fraction burned raster grid for extreme fire weather scenario (**CFB.tif**). Units= fraction between 0 and 1. Resolution = 40 m. Coordinate system= ETRS89 UTM 31N.
– Crown fraction burned raster grid for prescribed fire conditions (**CFB_Rx.tif**). Units = fraction between 0 and 1. Resolution = 40 m. Coordinate system= ETRS89 UTM 31N.

Fire transmission (TR) quantifies the number of residential houses exposed from fires ignited in a particular location. Values ranged between 0 to 417 structures. The raster is a continuous cover smoothed grid generated with a geospatial analysis from values attributed to the large fire (>100 ha) ignition locations. The result is highly dependent on fire spread duration, fire weather scenario, fuels arrangement and the location of valued assets on the landscape. Details of the geospatial data:

– Fire transmission to residential houses smoothed raster grid (**TR.tif**). Units = number of structures (residential houses) exposed. Resolution = 40 m. Coordinate system= ETRS89 UTM 31N.

## 2 Experimental design, materials and methods

We modeled with FlamMap [2] the 1) node influence grid (NIG); 2) crown fraction burned (CFB); and 3) fire perimeters required to assess fire transmission. The minimum travel time (MTT) algorithm [3] implemented in the program is used for fire growth modeling, and crown fire calculations are available with different methods [4], [5]. The MTT algorithm calculates a two-dimensional fire growth by searching for the set of pathways with minimum fire spread times from the cell corners at an arbitrary resolution [3]. We used landscape data and wildfire season fire weather conditions for fire modeling.

The landscape file is a regular grid containing spatial data for terrain (aspect, elevation and slope), surface fuels and canopy metrics (canopy height, canopy base height, canopy bulk density and canopy cover). We generated a 40 m resolution landscape file of 252,000 ha encompassing Bages County plus a 6 km buffer using ArcFuels [6]. The fire modeling domain was larger than the study area to account for incoming fires from neighboring vicinities. Required terrain and canopy metric data were respectively derived from a 5 m resolution digital elevation model and 20 m resolution forest cover biophysical data grids generated from low density airborne LiDAR (icgc.cat). We obtained the surface fuel model grid from the assignation of standard fuel models [7] to the habitat cartography of Catalonia considering species composition, vegetation cover percentage, average shrub height and species biogeographic locations on habitat polygon attributes (2° edition 2008/2012 version; mediambient.gencat.cat).

We used extreme fire weather conditions to emulate historical blow-up events overwhelming fire suppression capabilities in the study area. Specifically, we considered historical wildfire season 97th percentile conditions in terms of winds and fuel moisture content. In the study area the wildfire season is concentrated in the month of July, when large fires (>100 ha) account for 90% of burned area (1983 to 2014; mapama.gob.es). Hourly relative humidity, temperature, wind speed, wind direction, precipitation and solar radiation data from the Castellnou de Bages automatic weather station were used (1998 to 2016 records, U4 station reference; meteo.cat) to characterize extreme fire weather conditions with Fire Family Plus [8]. We obtained fuel moisture content data from 97th percentile ERC-G conditions [9], and 97th percentile wind speed corresponding to a predominantly southern direction (Table 1). Also, we simulated CFB for prescribed fire burn window conditions to assess the potential negative impacts of using fire to treat dense unmanaged forests. To carry out prescribed fire modeling we considered steady and persistent wind speed weather, and mild spring moisture content conditions. At these conditions, we can reduce surface and actively growing ladder fuels (i.e., shrubs and advanced regeneration), while deeper duff and higher soil moisture help to protect dominant tree root systems from fire damage.

**Table 1:** Fire weather data used in fire modeling in Bages County. Extreme fire weather conditions (97^th^ percentile) were used for large fire event modeling in the study area. Weather scenarios were generated with Fire Family Plus [8] using as reference 1998 to 2016 historic hourly data records from U4-Castellnou de Bages automatic weather station (meteo.cat). We considered the dominant wildfire season southern wind direction (180°) for fire modeling [10].

Fire weather conditions	**Fuel moisture content** (%)					**Wind scenario**
	1-h	10-h	100-h	Woody	Herbaceous	Speed (km h^−^^1^)
Extreme (97^th^ percentile)	7	8	11	60	20	24
Prescribed fire	12	13	15	100	60	10

**Fig. 1:**
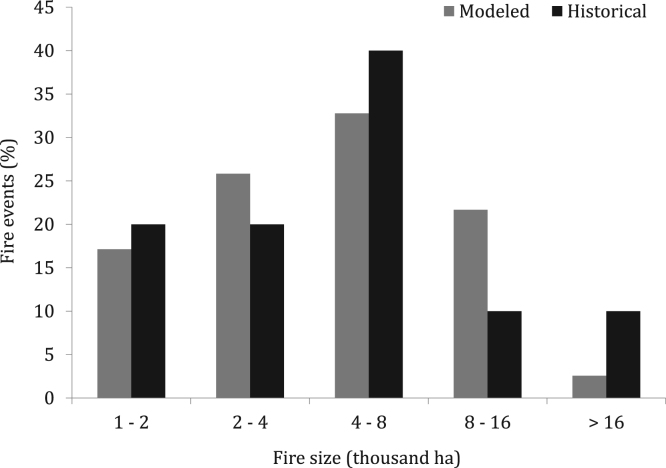
Historical and modeled fire size distributions in Bages study area. We considered historical large fire events above a 1000 ha threshold [10] to replicate fire size distributions with fire simulation modeling. The fire modeling duration was set to 8 h and weather conditions corresponded to a southern wind direction and 97^th^ percentile wind speed (Table 1). Historical and modeled average fire sizes were respectively 6,025 ha and 5,761 ha. For extreme fires burning for multiple days and spreading out of the study area we used the blow-up episodes burning inside the Bages County (i.e., > 10^4^ ha 4^th^ July 1994 Bages fire).

Fire modeling was conducted at 40 m resolution under constant fire weather conditions, fuel moisture content and wind (Table 1). In total, we simulated 10,000 fires from random ignitions within the fire modeling domain, which provided the same number of fire perimeters (shapefile polygons) attributed with their respective fire ignition coordinates. The fire modeling duration of 8 h resulted in average fire size and distribution that resembled historical large fire events (Fig. 1).

To generate the TR grid, we first intersected the large fire perimeters (*n*=6,816 fires >100 ha) with structure location centroids within the study area (*n*=23,633 structures), to then assign the number of structures exposed to wildfire to ignition locations [11]. Small fires do not substantially contribute to the burned area and were excluded to assess fire transmission. Finally, we used exponential kriging geostatistical methods (radius= 3000 m) to create a 40 m resolution smoothed TR grid from ignitions attributed with the number of exposed structures.
