# Supplementary material for: Wildfire spread, hazard and exposure metric raster grids for central Catalonia
Source: Data Brief. 2018 Jan 4;17:1–5. doi: 10.1016/j.dib.2017.12.069 (PMC5766747; doi:10.1016/j.dib.2017.12.069)
Supplement: Supplementary file 1 — Supplementary material [file mmc1.pdf]

CONFLICT OF INTEREST STATEMENT

Manuscript tittle: **Wildfire spread, hazard and exposure metric raster grids for central Catalonia**

Manuscript No.: **DIB-D-17-01245**

The authors whose names are listed immediately below certify that they have NO affiliations with or involvement in any organization or entity with any financial interest (such as honoraria; educational grants; participation in speakers' bureaus; membership, employment, consultancies, stock ownership, or other equity interest; and expert testimony or patent-licensing arrangements), or non-financial interest (such as personal or professional relationships, affiliations, knowledge or beliefs) in the subject matter or materials discussed in this manuscript.

The corresponding author and responsible of the research:

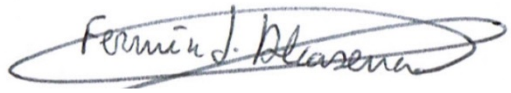

Fermín J Alcasena Urdíroz – Research Trainee Staff
Agriculture and Forest Engineering Department (EAGROF), University of Lleida,
Alcalde Rovira Roure 191, 25198 Lleida, Catalonia, Spain.
